# The Influence of Er and Zr on the Microstructure and Durability of the Mechanical Properties of an Al-Mg Alloy Containing 7 wt.% of Mg

**DOI:** 10.3390/ma17215295

**Published:** 2024-10-31

**Authors:** Kamila Limanówka, Sonia Boczkal, Monika Mitka, Elżbieta Szymańska, Joanna Hrabia-Wiśnios, Dawid Kapinos, Bogusław Augustyn, Renata Skrzyńska, Łukasz Grzebinoga, Paweł Czaja, Anna Góral, Tomasz Czeppe

**Affiliations:** 1 Łukasiewicz Research Network—Institute of Non-Ferrous Metals, Light Metals Center, 32-050 Skawina, Poland; sonia.boczkal@imn.lukasiewicz.gov.pl (S.B.); dawid.kapinos@imn.lukasiewicz.gov.pl (D.K.); lukasz.grzebinoga@imn.lukasiewicz.gov.pl (Ł.G.); 2 Institute of Metallurgy and Materials Science, Polish Academy of Sciences, 30-059 Cracow, Polandgoral.a@imim.pl (A.G.);

**Keywords:** Al-Mg alloys with high Mg content, micro-alloying elements, recovery, EBSD

## Abstract

Al-Mg alloys are characterized by permanent solid solution hardening and can additionally be work-hardened. The high mechanical properties of Al-Mg alloys with above-standard Mg content obtained after plastic deformation processes decrease over time. The addition of minor alloying elements like Er or Zr is an alternative method to improve the durability of mechanical properties and increase the strength of Al-Mg alloys due to densely and evenly distributed dispersoids being formed. In this paper, Al-Mg alloys with above-standard Mg content (7 wt.%) and Zr and Er micro-alloying elements and their influence on the microstructure and durability of the mechanical properties were examined. The cast ingots of AlMg7 alloys were characterized by a smooth surface without cracks. The plastic deformation process in a static compression test resulted in an about 60 HBW increase in the Brinell hardness of all the deformed alloys relative to casting. It was revealed that the addition of Er and Zr significantly improved the mechanical properties and durability of the mechanical properties of the Al-Mg after annealing. The addition of Er or Zr slightly restrained the decrease in the Brinell hardness after annealing but did not inhibit it.

## 1. Introduction

Aluminum–magnesium alloys with a high magnesium content are becoming even more significant in the world economy due to their functional properties, such as strength, corrosion resistance, and susceptibility to welding and plastic deformation. The strength of Al-Mg alloys increases with increasing concentrations of Mg [1,2,3,4]. These alloys can undergo work-hardening during plastic deformation processes and increase their mechanical properties. The solid solution hardening of these alloys results from the II type stresses induced in the solid solution by substituting larger Mg atoms for Al atoms [2]. According to the dependence published by Mondolfo [4], each 1% of magnesium dissolved in an aluminum solid solution increases the lattice parameter by 0.005 A. Jiang et al. [1] showed that with the increase in Mg content, the density of the shear bands crossing each other during cold rolling increased, leading to more efficient comminution of the initial coarse grains and the development of nano-sized cell structures and ultrafine grains, and a greater accumulation of dislocation density in the Al matrix. The recovery and recrystallization processes encountered during heat treatment decrease the mechanical properties of those alloys. One of the ways to eliminate the decrease in the mechanical properties is to introduce micro-alloying elements forming thermal stable dispersoids. It has been found that adding some rare elements, such as micro-alloying components (like Zr, Er, Sc, Y, Yb) can inhibit the recovery and recrystallization processes and improve the microstructure, mechanical properties, and thermal deformation stability of Al alloys [5,6]. Mochugovskiy et al. [5] showed that addition of 0.1 at.% Er and 0.1 at.% Zr to the Al-Mg alloy exhibits a more pronounced modifying effect. Moreover, they reported that in alloys containing Er and Yb, the stable grain structure remained up to a solidus temperature of 550 °C. The effectiveness of the fine dispersoids in controlling recovery and recrystallization processes depends on their size, distributions, and whether they are coherent with the aluminum matrix [7]. The introduction of Er and Zr to Al-Mg alloys can lead to the formation of densely and evenly distributed dispersoids coherent with the matrix, which can pin a grain boundary and increase the recrystallization temperature, as presented by Ding et al. [8]. Nevertheless, the Al-Mg alloys containing Er have been poorly studied for now, but the addition of Er and Zr as micro-alloying elements is a perspective to form a new alloy characterized by high and stable mechanical properties [9,10]. In their research, Xue et al. [11] have shown that the alloy Al-Mg-Zn-Er-Zr had fine precipitates Al_3_(Er, Zr), which during the two-stage homogenization, were evenly distributed in the Al matrix, increasing the hardness of the alloy. In turn, Pan et al. [12] showed that the introduction of Zr to an Al-Mg alloy containing 4 wt.% Mg improved recrystallization resistance by forming Al_3_Zr dispersoids with a tetragonal D023 structure. These particles were formed by decomposing a supersaturated solid solution of aluminum [13,14]. Since Zr has low solubility in Al, it requires low-temperature heat treatment to form metastable Al_3_Zr dispersoids. These dispersoids are hardy for dissolution and coarsen and can control the evolution of a grain structure during subsequent deformation processes [8]. Several other elements including Er can improve the mechanical properties by grain refinement and the precipitation of Al_3_Er dispersoids which are formed [15]. The formation of the face-centered cubic structure L12 of the Al_3_Er intermetallic phase improves the recrystallization resistance and the mechanical properties in Al-base alloys [10,16]. However, the excess of Er is ineffective by the formation of primary Al_3_Er at the grain boundaries. In the aluminum matrix, 0.2 wt.% Er can be dissolved [17]. Wen et al. [16] showed that the yield strength of Al-4.5 wt.% Mg with 0.2 wt.% Er is 50% higher than in the alloy without Er. However, they reported that simultaneously adding Er and Zr to the alloys can improve their mechanical properties and thermal stability due to the densely and evenly distributed Al_3_(Er, Zr) dispersoids, which are formed with face-centered cubic structure L12. Al_3_(Er, Zr) dispersoids and have a core–shell structure, where Er is a core with Zr enriched around the core [6]. Ding et al. [8] reported a delay in the recrystallization process in an Al-Mg alloy with 6.4 wt.% Mg and including Er and Zr. The annealing temperature of 250 °C on the investigated alloy demonstrated the alloy’s relatively high strength. The addition of Er and Zr increases the recrystallization temperature to at least 200 °C compared to Al-Mg alloys without Er and Zr. The recrystallization of Al-Mg alloys with the addition of Er and Zr begins in particle-free places; therefore, the Al_3_(Er,Zr) dispersoids could be fine and densely and evenly distributed [18].

Many of the experiments on Al-Mg alloys reported in the literature were carried out on alloys with Mg content not exceeding 6 wt.%. The literature data show that an increase in Mg content causes an increase in the mechanical properties of Al-Mg alloys. In this paper, Al-Mg alloys with above-standard Mg content (7 wt.%) were investigated. This Mg content was chosen because, based on numerous studies conducted in the Łukasiewicz Research Network—Institute of Non-Ferrous Metals, it is known that an increase in Mg content causes the cracking of ingots due to the presence of high stresses. The research focused on the possibility of inhibiting the process of decreasing the mechanical properties of Al-Mg alloys with above-standard Mg content after plastic deformation. It is well known that scandium is an effective element in inhibiting the recovery and recrystallization processes in Al alloys. This additive causes the formation of hard, fine, and evenly distributed particles that inhibit the movement of dislocations. However, its high price makes its commercial application difficult. Among the few possibilities for creating strengthening and coherent particles in aluminum alloys, Er is a promising micro-additive that can increase the mechanical properties of Al-Mg alloys and inhibit recovery and recrystallization. The addition of Zr results in the partial replacement of Er atoms by Zr atoms, and the lattice parameter of the Al_3_(Er, Zr) phase decreases, which is related to the reduction in the precipitate lattice mismatch to the Al matrix [19,20]. The novelty is to produce Al-Mg alloys with a high Mg content and micro-alloying elements and examine their influence on the durability of the mechanical properties of Al-Mg alloys. For this purpose, the Al-7 wt.% Mg alloys with micro-additions of Er and Zr after the static compression test and annealing were characterized.

## 2. Materials and Methods

**Figure 1:**
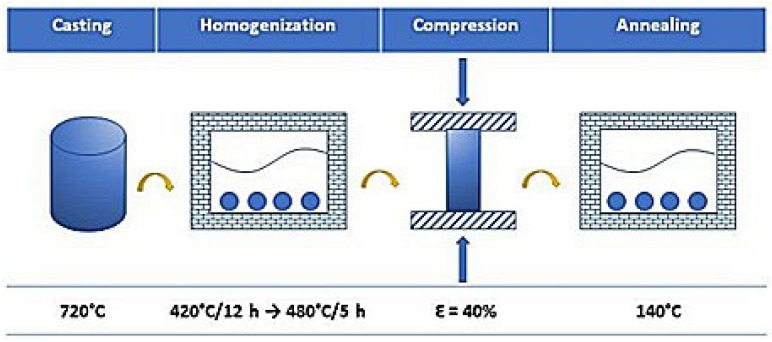
The scheme of the conducted research.

The research scheme was developed based on the technology used in the industry, as illustrated in Figure 1.

The base alloy was Al-7% wt. Mg. The addition of micro-alloying elements of Zr and Er to liquid aluminum was carried out at 690–740 °C. The micro-alloying elements were in the form of technically pure metals (Er) or mortars (Al-15 wt.%Zr). During the experiment, the appropriate temperature and the order of introducing the micro-alloying elements into the liquid metal were maintained. Experimental alloys were produced by melting about 2000 g of the charge in an induction furnace in a crucible of compacted aluminosilicate fibers. Then, it was cast at 720–740 °C into a steel casting mold heated to 200 °C. The shape of the mold allowed us to make castings in a horizontal position through the pouring systems and over the sample, guaranteeing that there were no casting defects, i.e., porosity and shrinkage cavities in the ingot. The weight of the castings, including the pouring system, was about 500 g. Cylindrical ingots with a diameter of 17 mm, a length of 170 mm, and a weight of about 100 g were cut from the castings. Samples for testing the microstructure and the Brinell hardness of the ingots, as well as for the static compression test, were taken from the center of the ingots. The chemical composition of the alloys was analyzed by optical emission spectrometry and is illustrated in Table 1. To eliminate interdendritical segregation, homogenization was performed in two steps, first at 420 °C for 12 h, then at 480 °C for 5 h. After the homogenization, the samples were submitted to the static compression test at room temperature with deformation equal 40%. Subsequently, the annealing was performed at 140 °C in 16, 48, 150, 465, and 1440 min to activate any recovery processes causing a decrease in the mechanical properties of the investigated alloys. After each stage of the investigation, the hardness of the alloys was measured using Duramin 2500E Brinell Hardness Tester (Struers, Copenhagen, Denmark) with a 2.5 mm ball under a load of 613 N. The microstructural characterization of the alloys was carried out by the light microscope Zeiss Axio Observer 7 Mat (Carl Zeiss, Oberkochen, Germany), scanning electron microscope Fei Inspect F50 (FEI Company, Hillsboro, OR, USA), and transmission electron microscope Fei Tecnai G2 (FEI Company, Hillsboro, OR, USA). The samples were polished using standard metallographic techniques and etched with Barker’s reagent for OM observations. The suitable surface finishes for EBSD and TEM analyses were obtained using electro-polishing techniques.

## 3. Results

**Figure 2:**
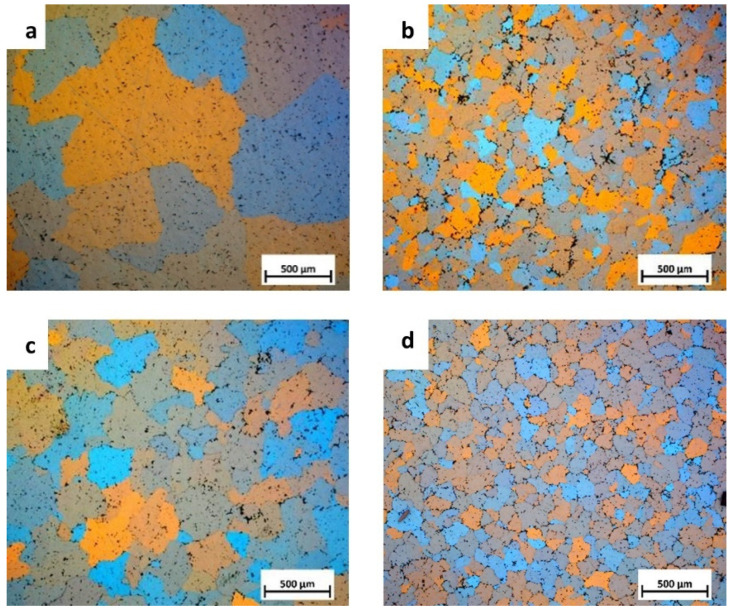
Optical micrographs of the grain structure for Al-7 wt.% Mg (**a**), Al-7 wt.% Mg with Zr (**b**), Al-7 wt.% Mg with Er (**c**), and Al-7 wt.% Mg with Er and Zr (**d**).

The influence of the introduced micro-elements was analyzed based on the microstructure examinations and the Brinell hardness measurements. Figure 2 shows the structures of the as-cast Al-Mg alloy and Al-Mg alloys with Er and Zr micro-alloying elements, etched using Barker’s reagent. The etching revealed grain boundaries to the effect that all of the samples have an equiaxed grain structure. The measurements of the average grain size in the as-cast alloy show that micro-alloying elements reduce the average grain size in Al-Mg alloys. The average grain size in the Al-7 wt.% Mg base alloy is 800 µm, the one containing 0.2 wt.% Zr is about 190 µm, the alloy with 0.25 wt.% Er is about 290 µm, and in the Al-7 wt.% Mg alloy with 0.2 wt.% Zr and 0.25 wt.% Er, the average grain size is about 220 µm.

**Figure 3:**
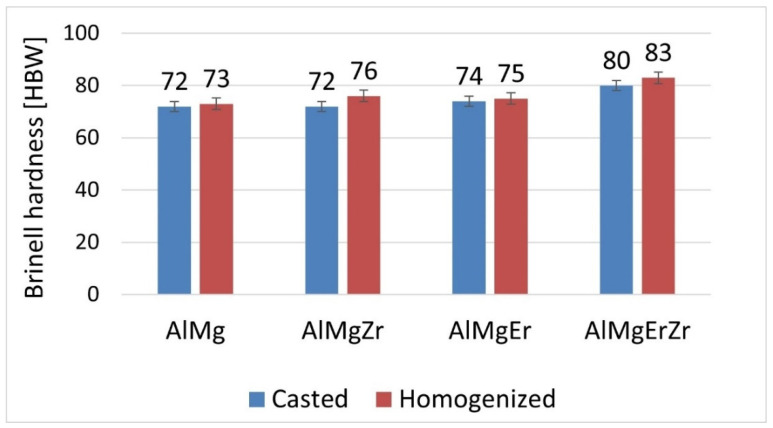
Brinell hardness of the ingots after casting and after homogenization.

Figure 3 shows that as-cast ingots had Brinell hardness in a range from 72 to 80 HBW, while homogenization resulted in a slight increase in the Brinell hardness in all of the tested alloys. The highest value of the Brinell hardness was measured in the sample with the simultaneous addition of Er and Zr.

**Figure 4:**
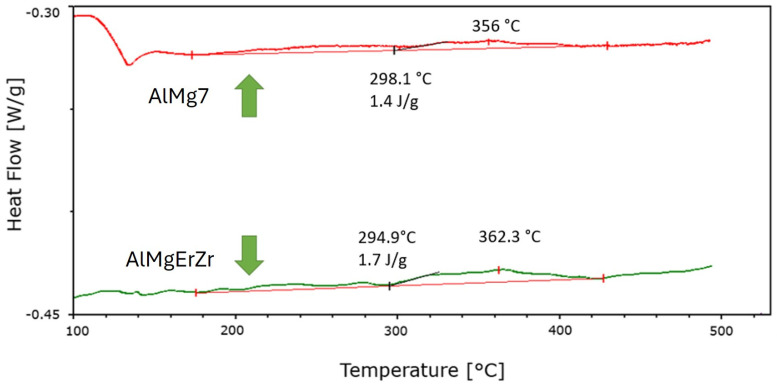
The DSC test of the AlMg7 alloy (red line) and the AlMgErZr alloy (green line).

All of the tested alloys revealed the increased Brinell hardness by approx. 60 HB after the compression test in comparison to cast ingots. The tested alloys were subjected to low-temperature heat treatment at 140 °C in order to activate the recovery processes that may occur in the tested alloys after a static compression test. The temperature was selected on the basis of differential scanning calorimetry tests (DSC), where the presence of an endothermic peak in the AlM7 alloy at a temperature of about 130 °C was observed, which may indicate the recovery processes taking place. This effect was negligible in the alloy of AlMg7 with the addition of Er and Zr. At higher temperatures, no distinct endothermic or exothermic peaks were observed, which could suggest processes occurring in both alloys, as shown in Figure 4.

**Figure 5:**
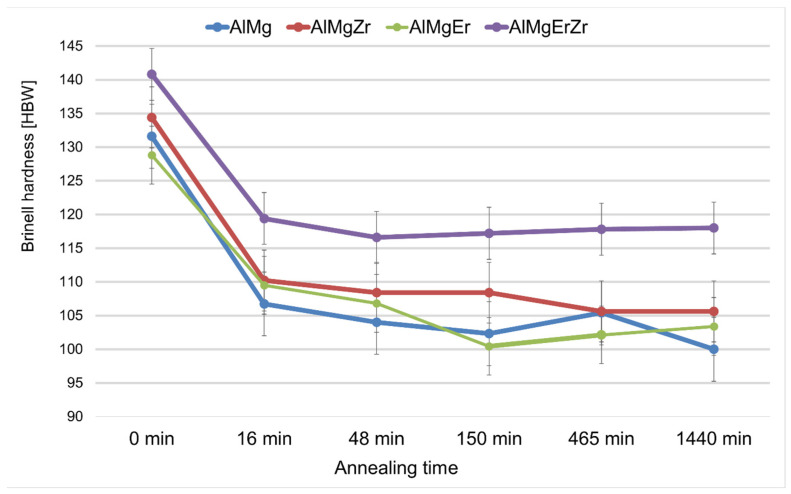
Brinell hardness of the samples after static compression test (0 min annealed) and after static compression test and annealing.

Because of the low-temperature heat treatment, the Brinell hardness decreased. The AlMg7, AlMgEr, and AlMgZr alloys showed the largest fluctuations in Brinell hardness during annealing. In the case of the AlMgErZr alloy, a decrease in Brinell hardness was measured after 16 min of annealing but in subsequent annealing times, its hardness was stable. The addition of Er and Zr inhibited the process of decreasing mechanical properties in the AlMg7 alloy after the compression test. The sample with the simultaneous addition of Er and Zr had the most stable hardness during annealing. In Figure 5, the Brinell hardness after deformation and the annealing all of tested alloys are presented.

**Figure 6:**
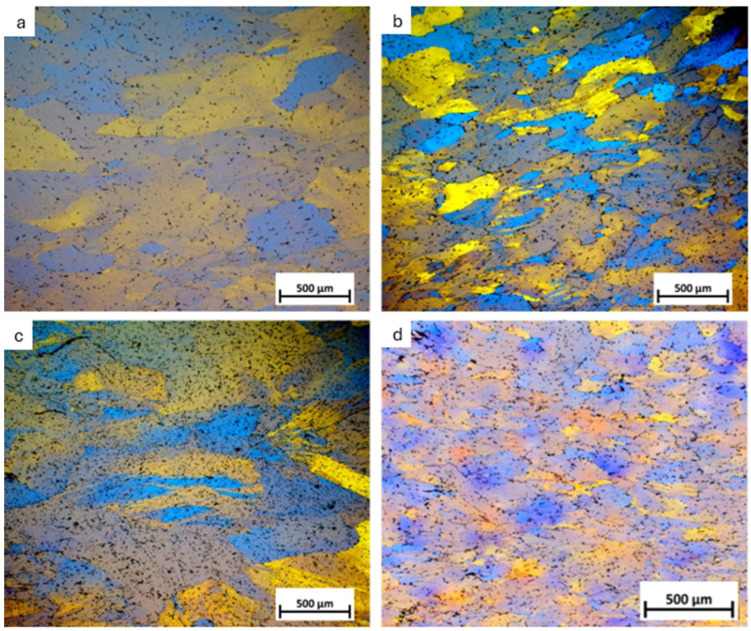
The LM microstructures after the compression test of the tested samples: AlMg7 (**a**), AlMgZr (**b**), AlMgEr (**c**), and AlMgErZr (**d**).

The deformation of the compressed samples is inhomogeneous. The deformation bands intersect to create the greatest deformation localization in the central part of the sample and smaller deformations in the outer part. Figure 6 shows light microscope images of the samples after the compression test. The observations were made on a cross-section in the middle part of the sample.

**Figure 7:**
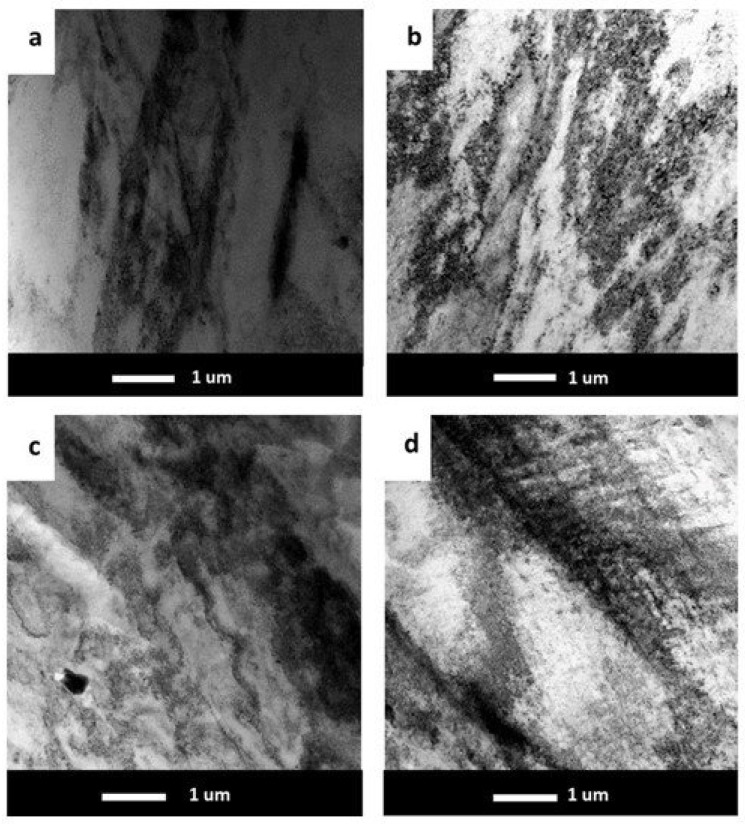
TEM bright field images of tested alloys after static compression test at room temperature for Al-7 wt.% Mg (**a**), Al-7 wt.% Mg with Zr (**b**), Al-7 wt.% Mg with Er (**c**), and Al-7 wt.% Mg with Er and Zr (**d**).

TEM investigations allowed us to show the evolution of the sample microstructure after the static compression test, as illustrated in Figure 7a–d. All of the deformed samples revealed a high density of dislocations. The samples containing micro-alloying elements had plenty of tangled dislocations.

**Figure 8:**
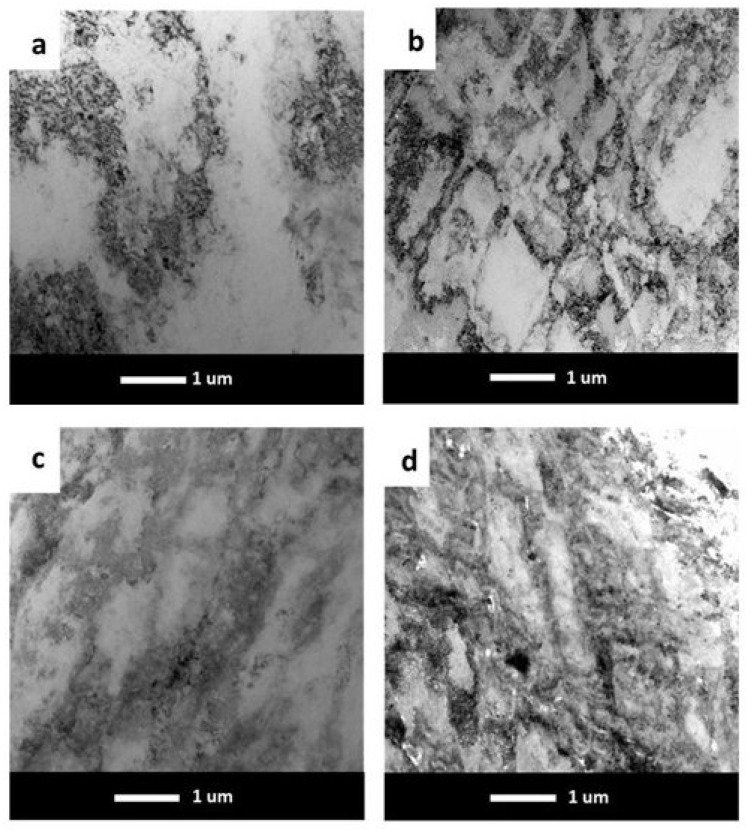
TEM bright field images of the alloys after annealing at 140 °C for 1440 min for Al-7 wt.% Mg (**a**), Al-7 wt.% Mg with Zr (**b**), Al-7 wt.% Mg with Er (**c**), and Al-7 wt.% Mg with Er and Zr (**d**).

With the process of reproducing the dislocation cells during the static compression test, the stress of the Al matrix was aroused. This stress performs the annihilation and rearrangement of dislocations, thereby declining the influence of work-hardening. The softening behavior increases with the increasing temperature. Figure 8a–d shows the microstructure after low-temperature heat treatment at 140 °C for 1440 min. In the Al-Mg alloy without micro-alloying elements and in the Al-Mg alloys with the single addition of Er or Zr, the density of dislocations after annealing decreased. In the alloy with the simultaneous addition of Er and Zr, the density of dislocations was still high after the compression test, as well as after annealing. Therefore, the lowest recovery and recrystallization effects were observed in the sample with the simultaneous additions of Er and Zr.

**Figure 9:**
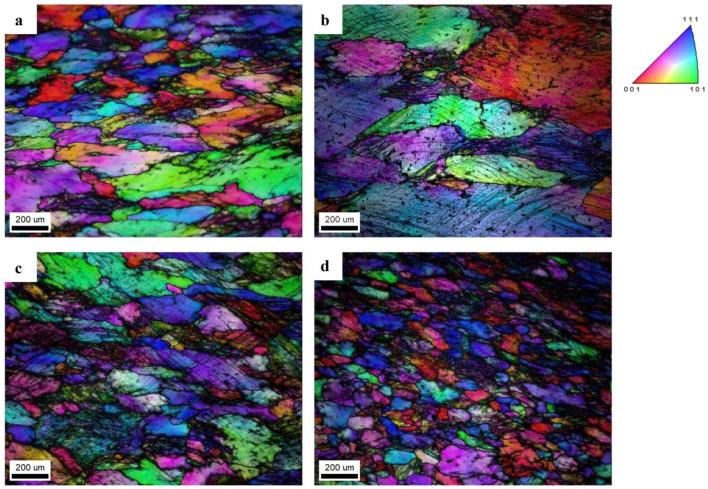
SEM-EBSD maps of deformed Al-7 wt.% Mg (**a**), Al-7 wt.% Mg annealed at 140 °C for 1440 min (**b**), deformed Al-7 wt.% Mg alloy with 0.2 wt.% Zr and 0.25 wt.% Er (**c**), and Al-7 wt.% Mg alloy with 0.2 wt.% Zr and 0.25 wt.% Er annealed at 140 °C for 1440 min (**d**).

To investigate the microstructure after the compression test and annealing, SEM EBSD maps of deformed structures were created, as illustrated in Figure 9. All of the tested alloys had numerous slip bands and strongly deformed grains after the compression test, with a high density of dislocations. The annealing of the Al-7 wt.% Mg alloy without micro-alloying elements caused grain growth (Figure 9b). The micro-alloying elements inhibited grain growth during annealing. The sample with the simultaneous addition of Er and Zr (Figure 9c,d) was composed of deformed grains with numerous slip bands after the static compression test, as well as after annealing.

**Figure 10:**
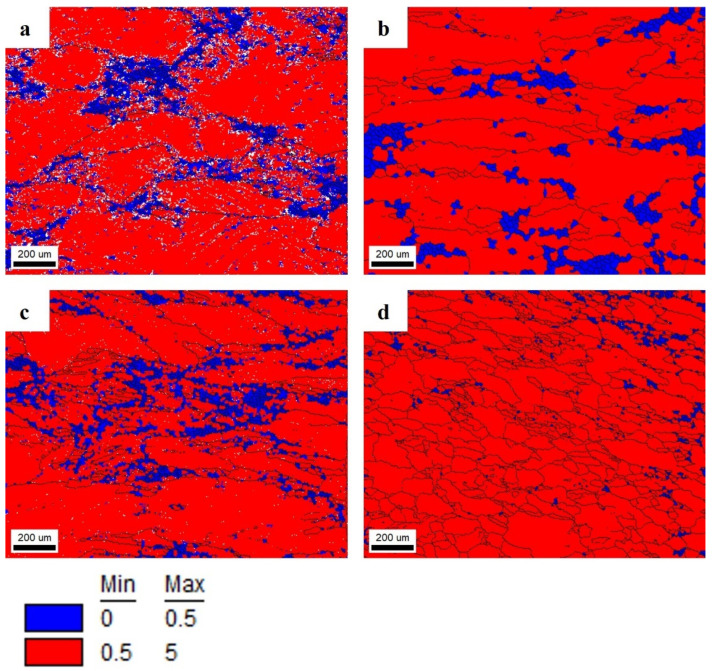
GAM distribution maps of alloys annealed at 140 °C for 1440 min: Al-7 wt.% Mg alloy (**a**), Al-7 wt.% Mg alloy with 0.2 wt.% Zr alloy (**b**), Al-7 wt.% Mg alloy with 0.25 wt.% Er alloy (**c**), and Al-7 wt.% Mg alloy with 0.2 wt.% Zr and 0.25 wt.% Er (**d**).

Conducted research showed that the addition of Er and Zr improves the mechanical properties and resistance to recrystallization of Al-Mg alloys. Figure 10 shows the grain average misorientation maps (GAM). The GAM value indicates the average misorientation between all neighboring pairs of points in the grain. It has been approved that low GAM values (below 0.5°) represent the restored areas by recovery or recrystallization [20]. Presented in Figure 10, GAM maps are colored in a two-colored gradient. The grains in blue have the smallest GAM values and indicate dislocation-free grains, and thus can be perceived as recrystallized grains. In contrast, the grains in red contain sub-grains, so they can be distinguished as uncrystallized grains. As a result, the most recrystallized structure after annealing at 140 °C for 1440 min was the Al-Mg alloy without micro-alloying elements (Figure 10a). For the Al-Mg alloy containing Er and Zr (Figure 10d), the recrystallized fraction is negligible.

**Figure 11:**
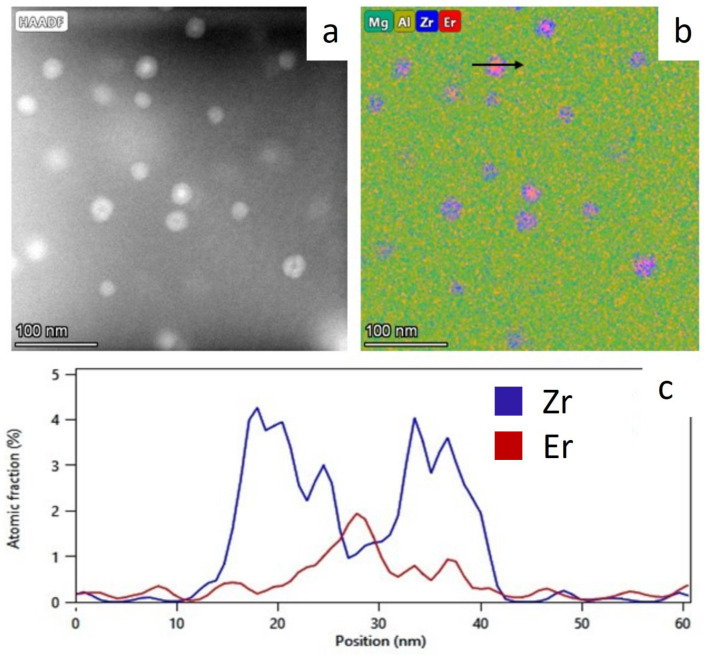
STEM image of the Al-7 wt.% Mg alloy with the addition of 0.25 wt.% Er and 0.2 wt.% Zr (**a**) and a corresponding chemical element distribution map with a marked black arrow (**b**) that indicates the EDS line profile of Er and Zr elements along the line (**c**).

An increased resistance to recrystallization is provided by the formation of the stable intermetallic precipitates. From the STEM image and EDS elemental map shown in Figure 11, it can be inferred that the precipitates with spherical shapes are densely and evenly distributed in the Al matrix. The size of the particles was found to be on the nanometer scale with a diameter of approx. 20 nm. The element distribution map confirms the presence of Er and Zr in the precipitates, where the Zr intensification is visible at the periphery of the precipitate, while inside the precipitate, the intensification of Er is evident. It may indicate that it is a core–shell Al_3_(Er, Zr) phase. Those precipitates can improve not only the room temperature strength but also the strength at higher temperatures.

**Figure 12:**
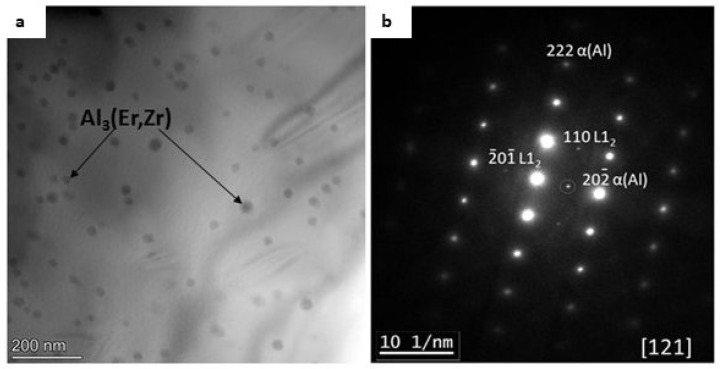
Bright field TEM image of the microstructure (**a**) and corresponding electron diffraction patterns from the selected particle (**b**) of the Al-Mg alloy containing Er and Zr.

Figure 12a shows the TEM bright field image of the Al-7 wt.% Mg alloy with the addition of 0.25 wt.% Er and 0.2 wt.% Zr and electron diffraction patterns from the selected area (Figure 11b). The presence of fine Al_3_(Er, Zr) dispersoids was confirmed. Figure 12b shows that those particles can be assumed as coherent with the Al matrix. Those Al_3_(Er, Zr) precipitates can inhibit the processes of dislocation movement in the Al-Mg alloys, inhibiting the recovery and recrystallization processes.

**Figure 13:**
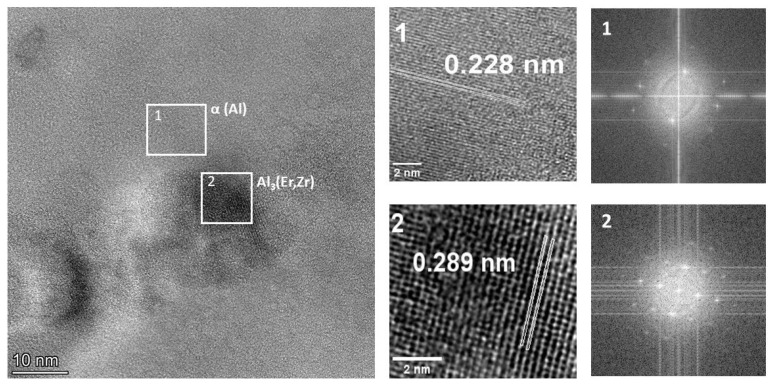
HRTEM image of the AlMgErZr alloy and Fourier transforms (FFTs) obtained from the Al matrix (1) and from the precipitate (2).

Observations of the AlMgErZr alloy using high-resolution transmission microscopy techniques allowed for the identification of Al_3_(Er, Zr) fine dispersoids and the determination of their relationships. Figure 13 shows a HRTEM image with a spherical Al_3_(Er, Zr) particle. Identification was based on the Fourier transform (FFT) method made of selected areas of the HRTEM image from the Al matrix (1) and the spherical precipitate (2).

The observations using HRTEM confirmed that the finely dispersed Al_3_(Er, Zr) precipitates are coherent with the Al matrix. Such particles provide resistance to the dislocations that surround them and create the so-called dislocation loops on them. A particle with a dislocation loop created on it is a more effective obstacle to the movement of subsequent dislocations.

## 4. Discussion

In the present work, the Al-7 wt.% Mg alloys with the micro-addition of Er and Zr were cast and the influence of the above-mentioned micro-alloying elements on the mechanical properties and microstructure was determined. In the research, the possibility of inhibiting the spontaneous decrease in the mechanical properties of AlMg alloys with high Mg content was analyzed. A compression test was performed to minimize the costs of the study. Promising alloys, characterized by stable mechanical properties, can be subjected to processes such as extrusion, forging, drawing, etc., to obtain products characterized by high and stable mechanical properties during use in the automotive, marine, food, and other industries. The addition of Er and Zr reduced the average grain size in the AlMg7 alloy by almost four times. Similar results were obtained by Pozdniakow et al. [21]; they showed significant grain refinements in the Al-Mg-Mn-Zr-Sc-Er alloy by the formation of Al_3_Er and ternary (Al, Mg, Er) phases. The Brinell hardness of AlMg, AlMgEr, AlMgZr, and AlMgErZr alloys after casting did not exceed 90 HBW (Figure 3). Those alloys, as a cast state, had permanent solid solution hardening with Brinell hardness from 72 to 80 HBW. In Al-Mg alloys, there are several strengthening mechanisms that exist, like solid solution strengthening by dissolving Mg in the Al matrix, precipitation strengthening, and work-hardening [20]. By comparing the Brinell hardness of AlMg, AlMgEr, AlMgZr, and AlMgErZr alloys after casting and the static compression test (Figure 5), it was shown that the static compression test increased the hardness by approx. 60 HBW for all of the tested samples. All alloys had numerous slip bands and strongly deformed grains (Figure 9), as well as a high density of dislocations (Figure 7a–d), after the static compression test. Min et al. [22] reported that hard-plate rolling Al–Mg alloys results in a high density of dislocation, while the density of dislocation increases dramatically with increasing Mg content. Unfortunately, the recovery and recrystallization processes occurring in Al-Mg alloys during heat treatment led to a reduction in high strength obtained in deformation processes. As a result of low-temperature heat treatment, the Brinell hardness in the tested alloys decreased. The highest decrease in the Brinell hardness was observed for the sample without micro-alloying elements. The addition of Er or Zr did not inhibit the process of decreasing the Brinell hardness of the tested alloys, but slightly slowed down its rate. In the analysis of the microstructure of AlMg, AlMgEr, and AlMgZr alloys, it has been shown that the density of dislocations after annealing dramatically decreased (Figure 8a–c) in comparison to the density of dislocations after the static compression test (Figure 7a–c). By contrast, the sample with the simultaneous addition of Er and Zr had the most stable hardness during annealing (Figure 5) and the density of dislocations was still high after the compression test (Figure 7d), as well as after annealing (Figure 8d). The decrease in the Brinell hardness for the AlMgErZr alloy was the smallest. The Brinell hardness drop was measured only after 16 min of annealing, while in subsequent annealing times, the hardness of this alloy was stable. It was shown that in the AlMg, AlMgZr, and AlMgEr alloys, there were changes in the Brinell hardness after individual annealing times, so the Brinell hardness of these alloys was not stable (Figure 5). This is because some grains are recrystallized and some are not, as shown in the GAM maps (Figure 10). In recrystallized grains, the hardness was lower than in deformed grains. Kotov et al. [5] reported that with the increasing concentration of Mg in Al-Mg alloys, dynamic recrystallization occurred, but the addition of Er and Zr formed fine dispersoids Al_3_(Er, Zr), which can increase recrystallization resistance in the tested alloy. The GAM maps confirmed this argument, as shown in Figure 10. The fraction of the low GAM value area is much larger in the Al-Mg alloy without micro-alloying elements, as well as in the alloys containing single additions of Er and Zr, after annealing at 140 °C for 1440 min (Figure 10a–c) than in the alloy containing both Er and Zr (Figure 10d). Song et al. [23] explained that this could be by forming Al_3_(Er, Zr) precipitates. These precipitates have a core–shell structure, comprising an Er-enriched core rimmed by a Zr-enriched shell. The diffusivity of Zr in Al is about 6.4 × 10^−24^ m^2^ s^−1^ at 573 K [24], while the diffusivity of Er in Al is about (4 ± 2) × 10^−19^ m^2^ s^−1^ [25], so it is much higher than the diffusivity of Zr in Al. Because of the high diffusivity, Er can nucleate, so Er concentration in the Al matrix decreases in comparison to Zr. Then, Zr begins to nucleate on the previously formed Er nuclei, so core–shell particles are formed. In this study, the core–shell structure of precipitates was shown in Figure 11, and the representative SAD patterns (Figure 12) have shown a characteristic lattice spot due to the presence of Al_3_(Er, Zr) dispersoids. Wen et al. [16] reported that the lower diffusivity of Zr can improve the stability of the precipitates. The stress accumulated in the alloys during the static compression test can be larger than the critical size to cut these particles [26]. Core–shell particles have a bigger size than non-core–shell particles. For dislocations, it is more difficult to cut core–shell precipitates than non-core–shell precipitates. In addition, Fu et al. [4] reported that Al_3_(Er, Zr) precipitates could pin the sub-grain boundaries and improve the recrystallization resistance. Therefore, the reason why AlMgErZr had the most stable Brinell hardness during low-temperature heat treatment was the formation of core–shell Al_3_(Er, Zr) dispersoids. As it is well known, Al_3_(Er, Zr) precipitates can inhibit the movement of dislocations by a pinning effect and it was shown (Figure 7d) that the AlMgErZr alloy had numerous slip bands and strongly deformed structures after the compression and annealing. Furthermore, Al_3_(Er, Zr) dispersoids inhibited grain growth during annealing. Similar conclusions were drawn by Gao et al. [27], who studied the Al-Mg alloy with the addition of Er, Sc, and Zr. They showed that the occurring in the grain boundaries, the Er-rich phase inhibited grain growth, which brings a significant strengthening of the grain boundary and the strengthening of the Orowan effect. The boundaries of the slip bands changed shapes because of the polygonization of the dislocations. The AlMg7 base alloy had a Brinell hardness of about 135 HB after compression, and its hardness gradually decreased with annealing time. The AlMgErZr alloy had a Brinell hardness of about 140 HB after the compression test, and its hardness decreased only by about 20 HB after 16 min, while after the subsequent annealing times, the hardness of this alloy was stable. The hardness of the AlMgErZr alloy decreased almost twice as much as the AlMg7 alloy after 1440 min of annealing, which means that the use of Er and Zr as micro-alloying elements inhibited the processes of loss of work-hardening. However, the single addition of Er and Zr did not have a significant influence on recrystallization resistance in Al-Mg alloys.

## 5. Conclusions

The static compression test has been conducted on Al-7 wt.% Mg with different combinations of Er and Zr additions, and the deformation equaled 40% for all of the tested samples. The effect of Er and Zr content on the mechanical properties’ stability after annealing was investigated by SEM EBSD, TEM, and the Brinell hardness methods. It has been shown that it is possible to produce an AlMg7 alloy characterized by stable mechanical properties after plastic processing by introducing Er and Zr. The main conclusions are presented below as follows:

- Micro-alloying elements resulted in a reduction in the average grain size in the ingots by almost four times. The alloys with micro-additions were characterized by a homogeneous structure comprising equiaxed grains. The grain refinement was caused by the presence of small precipitates of primary phases containing Er and Zr, which crystallize first in the liquid melt and constitute crystallization nuclei for the solid aluminum solution.
- Due to the solid solution and work-hardening after the static compression test, the Brinell hardness of all tested alloys increased by about 60 HB compared to the Brinell hardness of the ingots, which is the result of an increase in the density of dislocations.
- The simultaneous addition of Er and Zr to AlMg7 significantly improved the durability of the mechanical properties after annealing. It was caused by forming spherical Al_3_(Er, Zr) dispersoids with a diameter of about 20 nm, which inhibited the movement of dislocations during annealing. The pinning effect of Al_3_(Er, Zr) dispersoids supported sub-structure strengthening at an elevated temperature, resulting in the formation of an alloy characterized by stable mechanical properties. The fractions of recrystallized grains based on the GAM ≤ 0.5° parameter were about 5%. The Er and Zr single additives of AlMg7 slightly restrained the decrease in the Brinell hardness after annealing but did not inhibit the processes of its decrease after annealing. The fractions of recrystallized grains based on the GAM ≤ 0.5° parameter were about 20%. The highest decrease in Brinell hardness revealed the alloy without micro-alloying elements. This was caused by the microstructure reconstruction during annealing, which resulted in the dislocation annihilation. The fraction of recrystallized grains based on the GAM ≤ 0.5° parameter was about 35% for the AlMg7 alloy. These results correlated with a strengthening analysis based on the Brinell hardness measurement, as the highest decrease in hardness was demonstrated in AlMg7, AlMg7Zr, and AlMg7Er alloys. The share of the fraction after recovery and recrystallization based on the GAM parameter in the alloys was over three times higher than in the AlMgErZr alloy.

## Figures and Tables

**Table 1:** Chemical compositions of experimental alloys (wt.%).

Alloy	Chemical Composition [wt.%]									
	Fe	Si	Mg	Mn	Cr	V	Zr	Be	Er	Al
AlMg7	0.055	0.091	7.01	0.006	0.0003	0.002		0.002		Remainder
AlMgZr	0.051	0.053	6.96	0.002	0.0005	0.004	0.198	0.001		Remainder
AlMgEr	0.06	0.097	6.81	0.007	0.0004	0.002		0.002	0.12	Remainder
AlMgErZr	0.054	0.062	6.97	0.39	0.0008	0.004	0.204	0.002	0.27	Remainder

## Data Availability

The original contributions presented in the study are included in the article, further inquiries can be directed to the corresponding author.
